# Prediction models for prolonged air leak after pulmonary surgery: a systematic review and critical appraisal

**DOI:** 10.3389/fonc.2026.1899958

**Published:** 2026-08-12

**Authors:** Ning Du, Mengmeng Zhang, Xue Wang, Lijie Zhang, Yao Zhou, Chunxia Song

**Affiliations:** Chest Surgery Ward II, Shandong Provincial Hospital Affiliated to Shandong First Medical University, Jinan, Shandong, China

**Keywords:** lung cancer, prediction model, prolonged air leak, pulmonary surgery, systematic review

## Abstract

**Objective:**

This systematic review aims to comprehensively map, critically appraise, and synthesize the quality and performance of existing prediction models for prolonged air leak (PAL) following pulmonary resection, with a focus on their clinical utility and potential for translation.

**Methods:**

A systematic search was conducted in CNKI, Wanfang Data, VIP, SinoMed, PubMed, Web of Science, Embase, and the Cochrane Library, up to March 2026. Two researchers independently screened the literature, extracted data, and assessed the risk of bias in the predictive models. A qualitative, narrative synthesis of model characteristics and performance was performed. Results: A total of 26 models were included. Most studies had good applicability, but the overall risk of bias was high. The models showed a wide range of discriminatory power, with Area Under the Curve(AUC)values ranging from 0.644 to 0.914. Frequently identified predictors included pleural adhesions, forced expiratory volume in 1 second(FEV1), body mass index(BMI), age, smoking history, and gender.

**Conclusion:**

Existing predictive models exhibit considerable variability in reported discriminatory performance, with AUCs ranging from 0.644 to 0.914. However, the majority of these models are hampered by issues such as single-center development, insufficient external validation, incomplete calibration reporting, and methodological biases. The substantial heterogeneity observed across the reviewed studies precludes the generation of a single, reliable summary estimate of model performance. To enhance the clinical applicability and practical value of these predictive tools, future research should prioritize multicenter prospective studies, optimize variable handling and model validation strategies, and ensure comprehensive reporting of both discrimination and calibration.

**Systematic Review Registraton:**

https://www.crd.york.ac.uk/PROSPERO/home, identifier CRD420261435579.

Lung cancer represents a significant global public health challenge, ranking as the leading malignancy in incidence and mortality worldwide (1). Surgical resection is a cornerstone for managing lung cancer, especially in early-stage disease, where it significantly improves survival and prognosis. However, postoperative complications remain a frequent concern despite advances in surgical techniques and perioperative care. Among these, residual postoperative pleural space is notably common, with reported incidences ranging from 10% to 26% (2, 3).

Residual pleural space following pulmonary resection, often stemming from incomplete lung re-expansion and persistent air leakage, is a common finding. When this air leakage becomes prolonged, termed prolonged air leak (PAL), it poses a significant challenge in thoracic surgery. The unpredictable duration of air leaks can lead to prolonged chest tube placement and extended hospital stays, thereby increasing the risk of secondary postoperative complications (4, 5). Moreover, PAL is associated with higher medical costs and reduced patient satisfaction, ultimately placing an additional burden on healthcare systems. Therefore, effective prevention and management of postoperative PAL are crucial for optimizing surgical outcomes and improving healthcare efficiency. Given the substantial impact of PAL on both clinical outcomes and healthcare resource utilization, early risk stratification of PAL in postoperative patients is of critical importance. From a clinical perspective, accurate risk assessment enables surgeons to identify high-risk patients and implement targeted preventive strategies in a timely manner. From an economic standpoint, early identification of PAL risk facilitates rational allocation of medical resources and contributes to cost containment. However, the pathogenesis of PAL is multifactorial and complex, involving baseline pulmonary function, patient-related factors (such as age and BMI), lesion characteristics (including size and location), and perioperative inflammatory responses. This complexity makes early identification of PAL challenging when relying solely on clinical experience (6). To address this unmet clinical need, numerous predictive models for PAL have been developed in recent years by researchers worldwide (7–11). These models integrate demographic characteristics, laboratory indicators, and perioperative variables to estimate the risk of PAL occurrence. Although some models have demonstrated favorable discriminatory performance and have been presented as nomograms, risk scoring systems, or online prediction tools, their clinical utility remains uncertain. In particular, many existing models lack adequate external validation, and concerns persist regarding their predictive accuracy, risk of bias, and generalizability across different populations and clinical settings. Furthermore, the absence of comprehensive systematic reviews limits evidence-based selection and clinical implementation of these predictive models.

Therefore, this systematic review was conducted to comprehensively evaluate existing predictive models for PAL. Our primary objectives were: (1) to systematically assess the risk of bias and applicability of included predictive models using the Prediction Model Risk Of Bias And Screening Tool (PROBAST); and (2) to describe and synthesize key model characteristics and performance metrics, including discrimination (C-statistic/AUC) and calibration, through a structured narrative synthesis. By providing evidence-based insights into model quality and performance, this study aims to establish a foundation for the clinical selection of reliable prediction tools and offer robust guidance for the development of future high-quality predictive models. Furthermore, a discussion of common characteristics and challenges in model development is intended to inform and guide subsequent research efforts in this field.

## Methods

We conducted a systematic review with narrative synthesis following the Preferred Reporting Items for Systematic Reviews and Meta-Analyses (PRISMA) guidelines (12).The review was registered on PROSPERO (CRD420261435579).

### Search strategy

**Figure 1:**
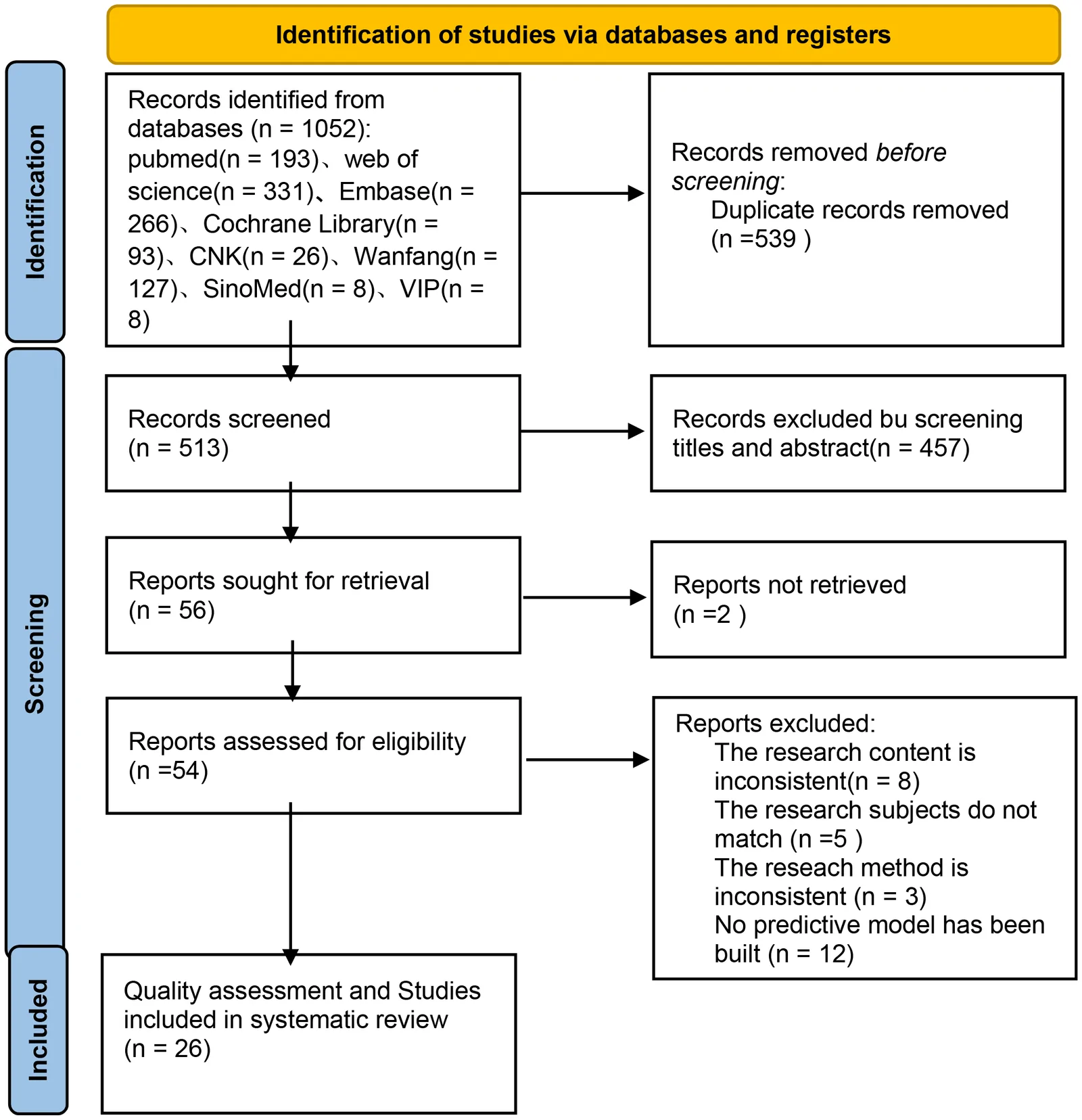
Search flowchart.

Searches were conducted in PubMed, Web of Science, Embase, Cochrane Library, China National Knowledge Infrastructure (CNKI), Wanfang Database, VIP and China Biomedical Literature Database. Additionally, this study systematically reviewed and included all eligible grey literature and reference lists. The search period spanned from the inception of each database to March 2026. We also performed manual searches of citations and reference lists from relevant articles. Duplicates were removed using both automated and manual strategies in EndNote. Using PubMed as an example, the search strategy is illustrated in **Figure 1**.

### Eligibility criteria

A systematic search adhering to the PICOTS framework (Population, Index Model, Comparative Model, Outcome, Timing, Setting) was employed to define study eligibility (12). Eligible studies included original research published in English or Chinese involving adult patients (≥18 years) undergoing lung resection for lung cancer or other lung diseases. The studies must have developed or validated at least one risk prediction model for PAL, with the outcome of interest being PAL occurrence. Predictions considered were those made pre-operatively, intra-operatively, or shortly post-operatively (prior to discharge) in inpatient thoracic surgery settings. Conversely, studies were excluded if they were non-original research (e.g., abstracts, reviews, commentaries), lacked full text or complete data, represented duplicate publications, analyzed risk factors without model construction, failed to report modeling processes, or were not published in English or Chinese. No comparative models were required for inclusion.

### Screening process and data extraction methods

Two independent reviewers (DN and SCX) extracted study and cohort characteristics using the CHARMS checklist (13), covering author, year, country, study type, sample size, modeling method, presentation format, variable selection, predictors, missing data handling, model performance, and validation. Any disagreements encountered during the screening or data extraction phases were resolved through discussion to achieve consensus. If consensus could not be reached, a third reviewer (MMZ) was consulted to adjudicate the final decision. For quantitative analysis, discrimination metrics (AUC/C-statistic with 95% CIs) and calibration metrics (goodness-of-fit P-value, calibration slope, Brier score) were extracted. Predictive factors were classified into three distinct categories: patient-related factors, surgical factors, and postoperative/intraoperative monitoring parameters. This classification framework aligns with approaches commonly adopted in systematic reviews of perioperative prediction models for thoracic surgery (6, 14).

### Risk of bias assessment

Risk of bias and applicability of included models were independently assessed by two investigators using the PROBAST tool (15, 16). Bias risk evaluation covered four domains: participants, predictive variables, outcomes, and analysis, each with multiple essential questions. Responses (“yes/probably,” “no/probably not,” “no information”) determined bias risk classification (low, high, or unclear). A domain was deemed high risk if any question received a “no/probably not” response; low risk required all questions to be “yes/probably.” Ambiguous overall judgments could arise from “no information” responses in some domains, even if others indicated low risk. To ensure consistency in the application of the PROBAST criteria, the two independent reviewers piloted the tool on three representative studies. Discrepancies identified during this piloting phase were discussed to calibrate their understanding. Subsequently, during the formal assessment, disagreements were resolved through structured consensus discussions. If consensus could not be reached, a third reviewer (MMZ) provided the final decision. All adjudicated items were meticulously documented to maintain transparency. Applicability was assessed similarly across participants, predictive factors, and outcomes.

### Outcomes

Prolonged air leak (PAL) was defined as air leak persisting beyond postoperative day 5, which is consistent with the definition adopted by the Society of Thoracic Surgeons (STS) General Thoracic Surgery Database and the current literature (17), lasting longer than 5–7 days (18).

### Statistical analysis

Anticipating substantial clinical and methodological heterogeneity across studies—as evidenced by the wide variation in PAL definitions, prediction timing, study populations, and resection extent—a narrative synthesis approach was planned. This strategy was selected to accommodate the inherent variability in study designs, populations, and outcome definitions, thereby enabling a structured description and comparison of model characteristics and performance. Formal meta-analysis was precluded because the observed variation in study characteristics was deemed too extensive to produce a meaningful pooled estimate.

## Results

### Selection process

The initial literature search yielded 1052 scientific articles. After reviewing the title and abstract of each article, we excluded those that did not conduct PAL risk prediction models, reducing the number of articles to 54. We conducted a full-text review and included 26 manuscripts that met the inclusion criteria (**Figure 1**).

### Study methodologies and study subjects

**Tables 1** and **2** present the specific characteristics of the included models. Among the studies included in the analysis, 17 models were based on single-center retrospective cohort studies, 6 models were based on multicenter retrospective cohort studies, and 3 models were derived from prospective cohort studies. Of the cohorts involved, 13 were located in China or Asia, 9 were located in Europe, and the remaining 4 were located in North America. PAL incidence rates ranging from 5.3% to 25.79%.

**Table 1:** Characteristics of the included models.

Author, Year	Location of study	Sample size (n)	Study type	Model presentation format	Definition of PAL (day)	Occurrence ratios for outcomes (%)	Number of predictors	Predictors ultimately included
Pan 2019	China	493	①	nomogram point scoring system	>5	10.8	8	age, smoking, FEV1/ FVC, pleural adhesion, stapling length , drainage ,BMI, right-side operation
Brunelli 2004	Italy	588	①	recurrence relation	>7	15.6	3	ppoFEV1, pleural adhesions,upper lobe resections.
Brunelli 2010	Italy	658	②	scoring system	>5	13	4	age >65 years,pleural adhesions,FEV1 < 80%,BMI < 25.5 kg/m2
Rivera 2011	French	30,926	②	rating formula (IPAL)	>7	6.9	9	gender, BMI, dyspnea score, pleural adhesions, lobectomy or segmentectomy, bilobectomy, bulla resection, pulmonary volume reduction, and location on upper lobe
Li 2022	china	2213	①	nomogram+web tools	>5	15.4	8	age, BMI, smoking history, FEV1% , surgical procedure, surgical range, operation side, operation duration
Attaar 2017	America	2317	①	nomogram	>5	8.6	7	FEV1%, smoking history, bilobectomy, higher annual surgeon caseload,previous chest surgery, Zubrod score >2, and interaction terms for right-sided thoracotomy and wedge resection by thoracotomy.
Omura 2024	Japan	494	①	scoring system (PPALS)	>5	5.9	4	FEV1% <60%, ANP <1 cmH2O, air leak flow >20 mL/min , pleural adhesion
Lee 2011	Canada	580	②	weighted scoring system	>7	14/18	3	pleural adhesions ( 2 points), FEV1 ( 1 per 10% below 100%), and DLCO( 1 per 20% below 100%).
Viti 2018	Italy	459	②	scoring system	>5	15.69	5	FEV1%< 86% (1.5 points), BMI <24 (1 point), active smoking (1.5 points), incomplete fissures (1.5 points), adhesions (1 point).
Gilbert 2016	Canada	325	③	Weighted scoring system	>7	8	5	male gender (1 point), BMI ≤25 (0.5 point), smoker(2 points), DLCO% <80% (2 points), MRC dyspnea score >1 (1 point).
Jin 2021	China	1511	①	Nomogram	>5	9.07	5	age, FEV1%, surgical type, surgical approach ,smoking history
Oh 2017	South Korea	779	①	SUM Variable Score	>5	18.8	4	male gender, BMI < 25.5 kg/m2, right side operation, pleural adhesion
Divisi 2023	Italy	6236	②	LR	>7	11.3	8	middle lobe resection ,ground glass opacity,decreased,DLCO/VA ratio, male sex, COPD, rightside surgery ,prolonged duration of surgery the need for adhesiolysis.
Seder 2019 PALS	America	52,198	②	scoring system(PALS)	>5	10.4	5	BMI≤ 25 kg/m2 (7 points), lobectomy or bilobectomy (6 points), FEV1≤ 70% (5 points), male sex (4 points), right upper lobe procedure (3 points)
Ma 2024a	China	872	①	Nomogram	≥7	7.0	4	thoracic adhesion, history of respiratory disease, time of operation, LNs, LMR
Qian 2024	China	2503	②	Nomogram	24h	11.35/10.46	5	age >70, FEV1% <80%, nodule size, benignity/malignancy, pleural adhesions (none, focal, diffuse)
Shintani 2022	Japan	57532/30967	②	LASSO regression equation	≥7	5.3	9	age, sex, BMI, comorbid interstitial pneumonia, smoking habits, FEV1, tumor histology, multiple lung cancer, tumor location
Pompili 2017	European	7523	②	scoring system(ESTS)	>5	9.9	3	male gender ,FEV1%<80% ,BMI<18.5 kg/m2
Galetin 2025	German	131	①	scoring system	>7	16	6	BMI,cumulative pack-years,immunosuppression,adhesiolysis,operation time, GS ≥ 6
Rodríguez 2015	Spanish	100	③	LR	≥5	10	2	preoperative PAL score,air flow at forced deep expiration
Yoon 2023	South Korea	325	①	cox regression equation	>5	18.2	3	flow at 3 POH (≤80 mL/min), operation time (≤220 minutes), and right middle lobectomy
Wang 2025	China	82	①	Nomogram	≥5	18.3	4	smoking history, FEV1%, pleural adhesions, VE/VCO2 slope
Ma 2024b	China	738	①	Nomogram	≥7	8.3	4	BMI, smoking status, time of surgery, pleural adhesions,ALI
Zhu 2026	China	209	①	LR	≥5	25.79	7	age≥70,BMI<18.5kg/m2,COPD,FEV1%<70%,FEV1/FVC≤70%, PEF<300 L/min, Pleural adhesions
Li 2023	China	539	③	Nomogram+Web Tools	>5	18.1	6	PNI, air leak in POD1, pleural adhesions, mean pleural pressure gradient
Wu 2017	China	359	②	regression equation	>7	14.2	6	BMI, gender, smoking history, FEV1%, pleural adhesions, upper lobe resection

NR, Not reported; IV, Internal Validation; EV, External Validation; -, Not applicable. ① Single-center retrospective cohort; ② Multicenter retrospective cohort; ③ Single-center prospective cohort; forced expiratory volume in 1 second(FEV1); forced vital capacity(FVC); predicted postoperative FEV1(ppoFEV1); carbon monoxide diffusing capacity(DLCO);prognostic nutritional index(PNI);body mass index(BMI);Chronic Obstructive Pulmonary Disease (COPD); lymphocyte-to-monocyte ratio (LMR); advanced lung cancer inflammation index (ALI);Peak Expiratory Flow(PEF); additional negative pressure(ANP); postoperative hour(POH); Postoperative day 1 (POD1); Goddard score (GS); Medical Research Council dyspnea (MRC);Qian et al. (2024) defined PAL as air leak persisting for 24 hours postoperatively (inability to remove the chest tube due to air bubbles), which differs from the >5-day or >7-day definitions used in the majority of included studies.

**Table 2:** Validation and performance of the included models.

Name	Modeling methods	Model performance	Validation method	Handling of missing data	Handling of Continuous data
Pan 2019	LR	CI =0.858(Bootstrap validation)	IV(Bootstrap)	complete case analysis	ROC cutoff value
Brunelli 2004	LR	NR;	IV (Bootstrap)	complete case analysis	NR
Brunelli 2010	LR(backward stepwise variable selection+AIC)	CI =0.71 (95% CI 0.65 -0.77)(D)	EV CI =0.69 (95% CI 0.59 - 0.77)	mean/mode Imputation	ROC cutoff value
Rivera 2011	LR(backward stepwise variable selection+AIC)	H-L test: (p=0.4); CI: 0.71 (95% CI 0.70—0.72)(D); calibration slope:1; Brier:0.060	EV H-L test: (p=0.24); CI: 0.69(95% CI 0.66—0.72); calibration slope:0.874; Brier:0.053	multi-step interpolation	included consecutively
Li 2022	LR	H-L test: (P=0.388); AUC=0.7315 (95%CI 0.6979–0.7651)(D); DCA	IV (Bootstrap) H-L test:(P=0.577); AUC= 0.7325(95%CI0.6743–0.7906)	NR	included consecutively
Attaar 2017	LR(backward stepwise variable selection)	H-L test: (P=0.735); nomogram discriminatory accuracy= 76% ; C statistic=0.759(95%CI, 0.72-0.79)(D); Calibrate Slope:1.000	IV (Bootstrap) CI=0.738	NR	ROC cutoff value,included consecutively
Omura 2024	LR(backward stepwise variable selection) PPALS	AUC= 0.819 (95% CI0.726–0.912)(D);	EV AUC= 0.823 (95% CI 0.718–0.929)	NR	ROC cutoff value
Lee 2011	LR+Weighted grading system	H-L test: (p > 0.2);	EV H-L test: (p > 0.2)	NR	Grading and Scoring
Viti 2018	LR	H-L test: (p = 0.971); AUC: 0.805(D).	IV(Bootstrap)	exclude variables with high missing values (>10%)	ROC cutoff value
Gilbert 2016	LR(forward stepwise multivariate regression analysis)+Weighted grading system	AUC: 0.8 (95% CI, 0.7–0.9)(D); sensitivity (83% [95% CI [ 58 - 96]) specificity (65% [95% CI [ 58 - 71]).	IV AUC: 0.8 (95% CI, 0.7–0.9); sensitivity :86% specificity :46% ;	complete case analysis	NR
Jin 2021	LR(forward stepwise multivariable logistic regression analysis)	H-L test: (p = 0.79); CI =0.70(D); Optimal cutoff value:12; sensitivity =56% specificity =68%	IV CI =0.77; H-L test: (p = 0.51);	complete case analysis	clinical staging
Oh 2017	LR	AUC: 0.819 ; positive predictive value:75.7%; negative predictive value:77.7%	None	complete case analysis	ordered classification
Divisi 2023	RF+LR++ 10 cross-validation	C-statistic=0.68(0.66–0.70)(D); Brier=0.095(0.09–0.10) Emax = 0.050;Eavg = 0.005	IV C-statistic=0.63(0.57–0.67) Brier=0.098(0.08–0.10) Emax = 0.142;Eavg = 0.019	KNN interpolation	scaling + centering
Seder 2019	LR++Weighted grading system	AUC = 0.644(Cross-validation); negative predictive value = 91%;positive predictive value = 19%;sensitivity = 30%; specificity =85%; correctly classifies = 79% .	IV(cross-validation)	complete case analysis	clinical cutoff value
Ma 2024a	LR	AUC=0.823(95% CI 0.768-0.879)(D,via bootstrap validation).	IV(Bootstrap )	complete case analysis	ROC cutoff value
Qian 2024	LR	AUC = 0.813(D); H-L test: (P = 0.79). sensitivity =81.7%; specificity =68.1% optimal cutoff value:68	EV CI = 0.833; L test: (P = 0.24). cross-validation	NR	ROC cutoff value
Shintani 2022	LASSO+LR+BIC	NR	EV AUC= 0.6895.	NR	grade classification
Pompili 2017	LR	NR	EV	mean/mode Imputation	ROC cutoff value
Galetin 2025	LR	AUC = 0.914(includingGS ≥ 6)(D); AUC = 0.864(expet GS)(D). AUC = 0.625 (GS only, Development)	None	exclude missing values	ROC cutoff value
Rodríguez 2015	LR	CI = 0.83(95%CI: 0.73–0.93)(D).	None	no missing items	included consecutively
Yoon 2023	ROC+Kaplan-Meier + Cox	AUC = 0.911 (95% CI: 0.864–0.958)(D). sensitivity = 88.9% specificity = 82.5%	None	exclude missing values	ROC cutoff value
Wang 2025	LASSO + LR	H-L test: (p = 0.986); AUC = 0.914 (95%CI:0.851-0.977)(D); sensitivity = 93.3% specificity = 74.6%	None	complete case analysis	ROC cutoff value
Ma 2024b	LR	AUC = 0.784 (95%CI:0.720-0.848)(D); Brier score = 0.063	IV(Bootstrap)	complete case analysis	ROC cutoff value
Zhu 2026	LR	H-L test: (P=0.340); AUC = 0.828 (95%CI:0.780-0.876)(D). sensitivity = 79.2% specificity = 83.6%	EV H-L test: (P=0.184) AUC =0.804[95%CI(0.762-0.841)]. sensitivity = 80.5% specificity = 84.7%	complete case analysis	clinical cutoff value
Li 2023	LR	H-L test: (P=0.7655); AUC = 0.9092(95%CI:0.868-0.951)(D).	EV H-L test: (P=0.7494) AUC = 0.918[95%CI(0.864-0.972)].	complete case analysis	included consecutively
Wu 2017	LR	NR	EV AUC = 0.886 (95%CI:0.835-0.937) sensitivity = 78.5% specificity = 93.2%	complete case analysis	clinical cutoff value

NR, Not reported; D, Development Set; IV, Internal Validation;; EV, External Validation; -, Not applicable.Receiver Operating Characteristic Curve(ROC); Area Under the Curve ; (AUC); Logistic Regression (LR).

### Predictors of the included models

Among patient-related factors, FEV1% (including ppoFEV1 and FEV1/FVC) (16 studies), pleural adhesions (15 studies), BMI (14 studies), smoking history (10 studies), and gender (8 studies), age (7 studies) were the most frequently reported predictors, see **Table 3**. Among surgical factors, operative time (5 times), surgical site/right-sided surgery (4 times), upper lobe resection/tumor location (4 times) and surgical approach (2 times) were relatively common. Among postoperative or intraoperative monitoring parameters, leak flow (3 times) was the most frequently reported parameter. It is worth noting that pleural adhesions, FEV1% (including ppoFEV1 and FEV1/FVC), BMI, age, smoking history, and gender were identified as the most common predictive factors, see **Table 4**.

**Table 3:** Included predictors.

Category	Predictor	Frequency	Total
Patient-related factors	Pleural adhesions	15	
	FEV1% / FEV1 / ppoFEV1 / FEV1/FVC	16	
	BMI	14	
	Smoking history	10	
	Gender	8	
	Age	7	
	DLCO	3	
	COPD	2	
	Dyspnea score	2	
	PEF	1	
	VE/VCO2 slope	1	
	Zubrod score	1	
	GS	1	
	Comorbid interstitial pneumonia	1	
	Immunosuppression	1	
	Cumulative pack-years	1	
	Ground glass opacity	1	
	LMR	1	
	ALI	1	
	PNI	1	
	Nodule size	1	
	Benignity/malignancy	1	
	Previous chest surgery	1	
	Tumor histology	1	
	Multiple lung cancer	1	
	Tumor location	1	
	History of respiratory disease	1	
	LNs	1	
			99
Surgical factors	Upper lobe resection / location	4	
	Surgical procedure (lobectomy,bilobectomy, )	4	
	Operation side / right side	5	
	Operation time	4	
	Surgical approach	2	
	Stapling length	1	
	Bulla resection	1	
	Pulmonary volume reduction	1	
	Annual surgeon caseload	1	
	Surgical range	1	
	Middle lobe resection	2	
	Adhesiolysis	1	
	Need for adhesiolysis	1	
			28
Postoperative/Intraoperative Monitoring Parameters	Air leak flow	3	
	ANP	1	
	Drainage	1	
	Mean pleural pressure gradient	1	
			6

forced expiratory volume in 1 second(FEV1); forced vital capacity(FVC); predicted postoperative FEV1(ppoFEV1); carbon monoxide diffusing capacity(DLCO); prognostic nutritional index(PNI);body mass index(BMI); Chronic Obstructive Pulmonary Disease (COPD); lymphocyte-to-monocyte ratio (LMR); advanced lung cancer inflammation index (ALI); Peak Expiratory Flow(PEF); additional negative pressure(ANP); postoperative hour(POH); Postoperative day 1 (POD1); Goddard score (GS); Medical Research Council dyspnea (MRC).

**Table 4:** Key predictors.

Key factors	Frequency
Pleural adhesions	15
FEV1% (including ppoFEV1, FEV1/FVC)	16
BMI	14
Smoking history	10
Age	7
Gender	8

forced expiratory volume in 1 second(FEV1);body mass index(BMI).

### Modelling method

The 26 reviewed studies predominantly employed logistic regression (LR) as the foundational modeling approach for PAL prediction, with multivariate LR being the most common method for identifying independent risk factors. To refine variable selection, stepwise regression (primarily backward) was frequently utilized, employing AIC or likelihood ratio tests for inclusion/exclusion (7, 19–21). Model stability and overfitting were addressed through bootstrap resampling (500–1,000 iterations) for variable assessment or internal validation (7, 22–24). For high-dimensional data, LASSO regression facilitated variable selection via regularization (25, 26). Machine learning techniques included Recursive Feature Elimination (RFE) combined with 10-fold cross-validation for predictor screening (27). To improve clinical utility and visualization, nomograms were adopted (19, 23, 28, 29) translating regression coefficients into intuitive scoring tools. Weighted scoring systems were also used to create simplified risk scores based on proportional coefficients (20, 21, 30).

### Predictive performance and validation

The discriminatory performance (C-index or AUC) of the 26 PAL prediction models ranged from 0.644 to 0.914. The lowest reported C-index/AUC on the development set was 0.644 (Seder (31)), with the highest being 0.914 for a combined model (Galetin (21)). Sensitivity and specificity were reported in some studies. Of the 26 models, 10 underwent external validation using independent centers, different time periods, or treatment groups (7, 11, 20, 25, 29, 30, 32–35). 11 models reported internal validation, with the bootstrap method being common (9, 19, 22–24, 27, 28, 31, 36–38). Cross-validation was primarily used in machine learning studies (10-fold by Divisi (27) and repeated random split by Seder (31)), with training-test splits also employed (Li (28), Divisi (27)). 5 models lacked any form of validation (21, 26, 39–41). Externally validated models exhibited superior generalizability, while bootstrap and cross-validation represent mainstream internal validation techniques. A detailed breakdown of validation methods and performance metrics for each model is provided in **Table 2**.

### Handling of missing data and continuous variables

A considerable heterogeneity in methods for managing missing data and continuous variables was observed across the 26 PAL prediction model studies. Most studies either failed to explicitly describe their missing data handling strategies or utilized complete-case analysis. More standardized approaches were adopted by a few: Rivera (7) employed multiple imputation for dyspnea and ASA scores with high missing rates, while Brunelli (22)andViti (36)used mean/mode imputation for variables with <5-10% missingness. Divisi (27) applied K-nearest neighbors (KNN) imputation prior to machine learning. Some studies excluded variables exceeding a 5-10% missing rate (34, 42).

For continuous variables, the majority converted them into binary or ordinal categories using ROC or clinical cutoffs (e.g., FEV1% < 80%, BMI < 25.5 or < 18.5 kg/m², age > 65 years). Few studies retained continuous variables (e.g., BMI, dyspnea scores, pleural pressure gradient) to preserve original information. Shintani (25)used categorical approaches for age (in 5-year increments) and %FEV1 (in 10% increments), while Divisi (27)scaled and centered variables within a machine learning framework. Overall, current PAL prediction models largely rely on rudimentary missing data handling, with continuous variables typically categorized by truncated values. Studies preserving continuous variables or employing advanced interpolation methods remain scarce.

### Risk of bias and applicability assessment

**Figure 2:**
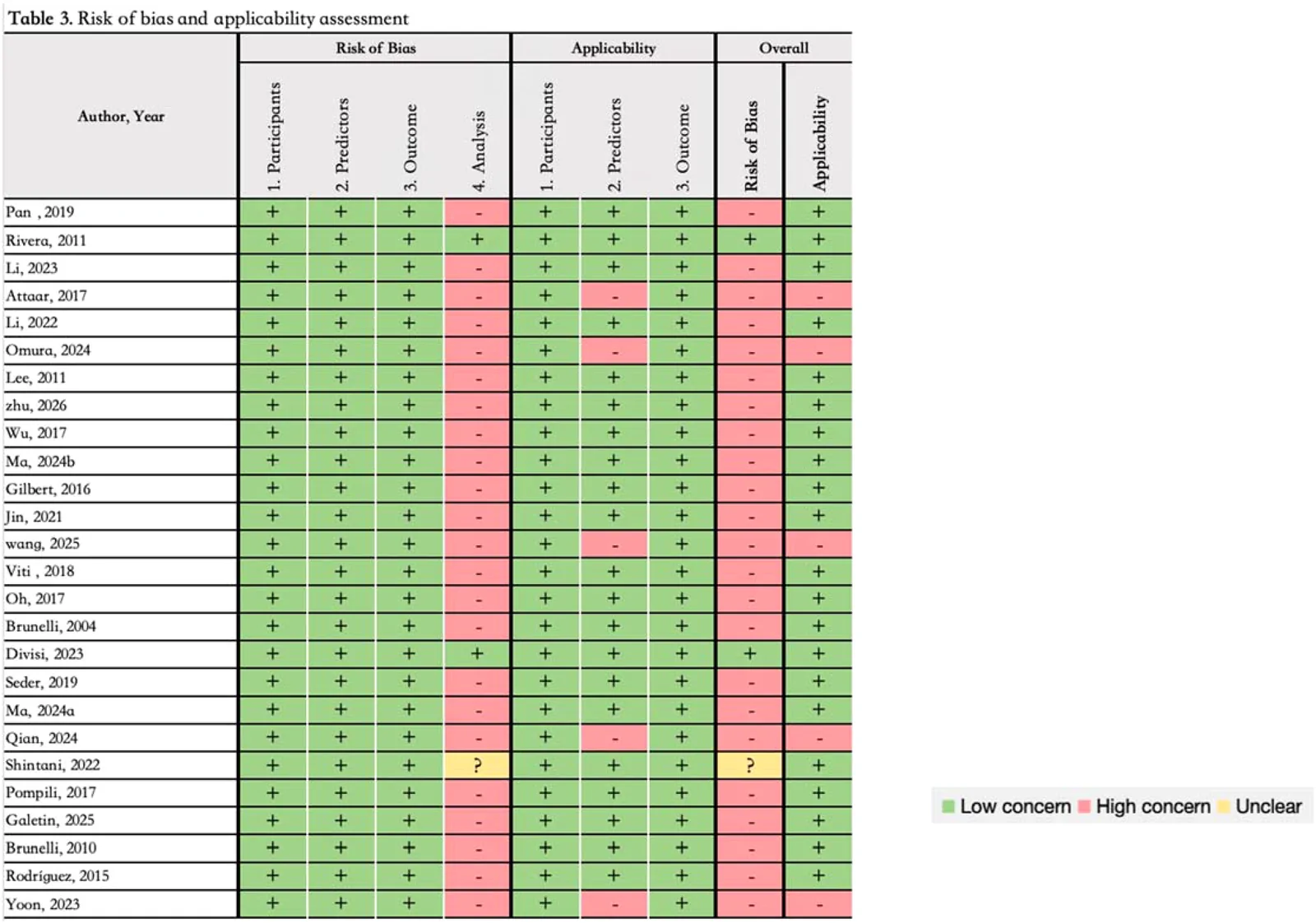
Bias risk assessment for all selected studies.

Using the PROBAST tool, 26 included PAL prediction model studies were evaluated for risk of bias and applicability. The two reviewers achieved consensus on all PROBAST domain-level assessments after structured discussion; disagreements—which occurred infrequently—were resolved through consensus or third-reviewer adjudication. Overall, Two studies were categorized as low risk, one study was unclear, and 23 studies were categorized as high risk (**Figure 2**, **Table 5**). Regarding applicability, 21 studies demonstrated high overall applicability due to well-aligned populations, predictors, and outcomes. 5 studies had low applicability, primarily due to requiring specialized equipment or having differing outcome definitions (e.g., extubation timing, not PAL occurrence). Conversely, limitations in high-risk studies comprised insufficient sample size (EPV < 10), lack of external validation, unclear missing data handling, and non-standard PAL definitions.

**Table 5:** Risk of bias ratings of all study developments and external validations using PROBAST.

Author, year	Participants	Predictors	Outcome	Analysis	Overall
Pan 2019	+	+	+	–	–
Rivera 2011	+	+	+	+	+
Li 2023	+	+	+	–	–
Attaar 2017	+	+	+	–	–
Li 2022	+	+	+	–	–
Omura 2024	+	+	+	–	–
Lee 2011	+	+	+	–	–
Zhu 2026	+	+	+	–	–
Wu 2017	+	+	+	–	–
Ma 2024b	+	+	+	–	–
Gilbert 2016	+	+	+	–	–
Jin 2021	+	+	+	–	–
Wang 2025	+	+	+	–	–
Viti 2018	+	+	+	–	–
Oh 2017	+	+	+	–	–
Brunelli 2004	+	+	+	–	–
Divisi 2023	+	+	+	+	+
Seder 2019	+	+	+	–	–
Ma 2024a	+	+	+	–	–
Qian 2024	+	+	+	–	–
Shintani 2022	+	+	+	?	?
Pompili 2017	+	+	+	–	–
Galetin 2025	+	+	+	–	–
Brunelli 2010	+	+	+	–	–
Rodríguez 2015	+	+	+	–	–
Yoon 2023	+	+	+	–	–

+ Positive rating, low risk of bias rating.

- Negative rating, high risk of bias rating.

? Unclear risk of bias rating.

EV – indicates external validation.

### Narrative synthesis of model performance

The included prediction models represent three fundamentally distinct categories based on their temporal window of application, each serving a unique clinical purpose: Preoperative models (n=4), exemplified by Gilbert (37), Jin (9), Pompili (30), and Brunelli (20), rely exclusively on pre-existing patient characteristics and lung function data to support patient selection and preoperative counseling, facilitating risk-benefit discussions prior to surgical intervention; reported AUC values for this category ranged from 0.644 to 0.858. Intraoperative models (n=17) incorporate variables known only at the time of surgery, such as pleural adhesion status, surgical approach, and operative time, aiding intraoperative decision-making regarding the application of prophylactic sealants or reinforcement materials; their reported AUC values ranged from 0.68 to 0.914. Early postoperative models (n=5) leverage immediate drainage data, including real-time air leak flow (Omura et al., 2024, at ANP ≤1 cmH_2_O), POD1 air leak [Li (29)], or flow at 3 postoperative hours [Yoon (41)], to support chest tube management and discharge planning; reported AUCs for these models ranged from 0.819 to 0.911. The models demonstrated a wide range of discriminatory power, with reported AUC values from 0.644 to 0.914. The best-performing models in terms of discrimination often incorporated intraoperative or early postoperative parameters such as real-time air leak flow [Omura (32)] or POD1 air leak [Li (29)], achieving AUCs above 0.90. In contrast, models relying solely on preoperative variables, such as the PALS score [Seder (31)], showed more modest discrimination (AUC 0.644). Externally validated models, such as the IPAL index [Rivera (7)], demonstrated consistent but moderate performance (AUC 0.69), highlighting the challenge of maintaining predictive accuracy across diverse populations.

The evaluated predictive models demonstrated a considerable range in discriminatory power, with reported AUC values spanning from 0.644 to 0.914. Notably, models incorporating intraoperative or early postoperative parameters consistently achieved superior performance, as evidenced by the high AUCs for real-time air leak flow (Omura (32), AUC = 0.819) and postoperative day 1 (POD1) air leak quantification (Li (29), AUC = 0.909). Four models surpassed a benchmark AUC of 0.90: Galetin (21) (AUC = 0.914) incorporating the Goddard score; Yoon (41), (AUC = 0.911) utilizing postoperative air leak flow; Wang (26) (AUC = 0.914) combining VE/VCO2 slope with traditional predictors (although developed in a small sample with limited validation); and Li (29) (AUC = 0.909) integrating PNI with POD1 air leak. In contrast, models relying exclusively on preoperative variables yielded more modest discriminatory performance, exemplified by the PALS score (Seder (31), AUC = 0.644). External validation results showed variability, with the IPAL index (Rivera (7), external AUC = 0.69) and the updated Brunelli score (Brunelli (20), external AUC = 0.69) exhibiting moderate and consistent performance, while the PPALS score (Omura (32), external AUC = 0.823) performed better in external cohorts. Calibration assessment was infrequently conducted, and among the limited studies reporting it, performance varied, with some models showing adequate goodness-of-fit (e.g., Rivera (7), 2011, Hosmer-Lemeshow test p = 0.4) and others lacking relevant calibration statistics, precluding a comprehensive evaluation of their predictive accuracy across different risk strata.

### Calibration assessment of included models

Calibration, defined as the concordance between predicted risk and observed outcomes, was assessed in a minority of the included models. Of the 26 included models, approximately 10 (38.5%) reported some form of calibration assessment, most frequently the H-L goodness-of-fit test. The results of the H-L test exhibited considerable variability: several models demonstrated adequate calibration with non-significant p-values (e.g., Rivera (7), p=0.4; Attaar (19), p=0.735; Li (28), p=0.388; Jin (9), p=0.79; Qian (34), p=0.79; Viti (36), p=0.971; Wang (26), p=0.986; Zhu (11), p=0.340; Li (29), p=0.7655;Lee (33), p > 0.2), suggesting acceptable agreement between predicted and observed risks within their respective development populations. However, it is crucial to note that the H-L test is known to be underpowered in smaller sample sizes and may fail to detect clinically meaningful miscalibration; therefore, its non-significant results should be interpreted with caution.

## Discussion

The significant and persistent challenge of postoperative PAL in thoracic surgery is underscored by our systematic review. Our comprehensive search of both Chinese and English databases yielded a substantial body of evidence, encompassing 26 studies that developed or validated predictive models for PAL. The wide range of reported PAL incidence, from 5.3% to a concerning 25.79% across these studies, unequivocally highlights the ongoing difficulty in its prevention and management, re-emphasizing PAL as a critical complication in thoracic surgery.

This systematic review found that while PAL predictive models generally demonstrated variable discriminatory ability (with AUCs ranging from 0.644 to 0.914), significant concerns impact their reliability and generalizability (27, 31, 40). Key limitations include: (1) Predominant reliance on retrospective data and its inherent biases, limiting generalizability. (2) High risk of overfitting due to insufficient sample size (EPV < 10 in 60% of studies), likely inflating reported AUCs and leading to decision-making bias. (3) Critically, only 10 studies performed limited external validation, exacerbating overfitting risks in diverse clinical settings. (4) Calibration assessment was infrequently and incompletely reported. While many models included some evaluation (commonly the H-L test), few provided rigorous metrics like calibration slopes or Brier scores. (5) Some studies had limited clinical applicability due to specialized equipment or procedural requirements. (6) Improper data handling, including unvalidated categorization of continuous variables and missing data deletion, resulted in information loss and reduced model robustness. Future research should focus on simplified models with accessible variables and robust validation.

The majority of the 26 PAL predictive model studies relied on single-center data, with only 10 utilizing multicenter datasets (five from existing databases (7, 25, 27, 30, 31) and the rest from individual hospital records). This reliance on diverse data sources limits model generalizability, highlighting a need for large-scale, multicenter trials with standardized data entry. Furthermore, there was a significant underutilization of unstructured data; only 4 studies extracted information from medical imaging (21), digital drainage systems (31, 32, 41) likely due to challenges in acquisition, processing, and analysis. Future research must prioritize large-scale, multicenter studies with standardized data collection and structured processing of EMR data to build a foundation for accurate PAL risk prediction models.

LR remains the predominant modeling approach for PAL prediction, though advanced techniques like RFE, LASSO regressionwere also employed (**Table 2**). While machine learning offers potential accuracy gains by capturing complex interactions, its “black-box” nature hinders clinical translation due to limited interpretability (42). Therefore, balancing predictive accuracy with interpretability is crucial. Recent trends favor robust development techniques, including LASSO regularization, nomograms, Bootstrap resampling, and cross-validation. For model application, nomograms and online calculators are common for clinical decision support, but direct integration into clinical workflows is underexplored. Critically, many models lack adequate testing for clinical validity, service impact, or economic outcomes; future research must include health economic evaluations and demonstrate tangible clinical impacts for wider implementation.

Our systematic review identified several common predictors for postoperative PAL: pleural adhesions, reduced FEV1%, BMI, advanced age, smoking history, and gender. Notably, BMI showed an inverse correlation with PAL incidence, potentially due to better tissue healing in obese patients (19). Smoking impairs collagen production and wound healing (43). Advanced age contributes to elevated risk via lung tissue fragility, reduced healing capacity, comorbidities, and declining respiratory muscle function. Gender emerged as a robust independent predictor, included in approximately 65% of scoring systems and 55% of LR models, consistently confirming its significance. Gender emerged as a frequently identified predictor, appearing in approximately one-third of the reviewed models. Meta-analyses suggest male sex is associated with a modestly increased risk of PAL (44). However, the strength of this association varies, with some large database analyses reporting male sex as a significant predictor while others find only borderline significance (6). It should be considered in conjunction with more robustly supported predictors such as pleural adhesions, impaired pulmonary function, and smoking history. However, the strength of this association demonstrated variability across the included studies. Poor lung function (low FEV1, FEV1/FVC, DLCO; COPD/emphysema comorbidities) is strongly associated with PAL, likely due to impaired tissue healing capacity (45); Furthermore, preoperative CT-based quantification of emphysema has been identified as an independent predictor of PAL (46). Pleural adhesions significantly increase PAL risk (P < 0.001) by complicating dissection, causing tissue damage, and leading to postoperative complications like effusion and increased pleural pressure (6). Early identification of high-risk individuals using predictive models and targeted interventions is critical.

A notable pattern identified in this review is the apparent inverse correlation between sample size and reported AUC, where smaller studies tended to report higher discrimination. For instance, the four models achieving AUC >0.90 were all developed in single-center studies with limited external validation: Wang (26) et al. (2025, n=82), Li (29) et al. (2023, n=539), Yoon (41) et al. (2023, n=325), and Galetin (21) et al. (2025, n=131). In contrast, larger and more methodologically robust studies, such as Rivera (7) et al. (2011, n=30,926) and Divisi (27) et al. (2023, n=6,236), reported more modest AUCs of 0.71 and 0.68, respectively. This observed trend is consistent with overfitting, a well-recognized phenomenon in prediction model development wherein limited sample sizes and an inadequate number of EPV can lead to overly optimistic performance estimates within the development dataset. Noting that while we applied the PROBAST-recommended EPV < 10 threshold for risk of bias classification, the field is moving toward more sophisticated sample size estimation approaches, and future PAL prediction model development studies should consider the Riley (47) et al. framework.

11 studies reported internal validation, while 16 studies lacked external validation. This suggests that the currently constructed models may exhibit good predictive ability on their original research data but have unclear generalization ability and reliability in different populations. Consequently, their clinical translation is difficult to support, and a high degree of application bias is indicated. Therefore, it is recommended that future research strictly adhere to the TRIPOD and PROBAST guidelines. External validation should be systematically conducted, and online assessment tools developed to further improve model reliability and clinical utility, ultimately reducing clinical workload. Furthermore, calibration reporting is severely lacking, with existing evidence largely confined to the AUC. Since AUC only reflects ranking ability and cannot demonstrate the consistency between predicted probabilities and actual risks, the absence of calibration plots, calibration slopes, and Brier scores leaves the reliability of models in real-world scenarios unclear. Therefore, future studies should uniformly report AUC, calibration plots, calibration slopes, decision curves, and Brier scores.

Key limitations of this review include the exclusive inclusion of English and Chinese literature, the substantial study heterogeneity that precludes the identification of a single optimal model, and the inherent challenges in applying the PROBAST criteria to machine learning-based prediction models, where certain signaling questions may not be directly applicable. Future research must prioritize large-scale, prospective, multicenter studies with standardized definitions and reporting, leveraging EHR data and data mining, adhering strictly to PROBAST and TRIPOD criteria, and balancing performance with interpretability for enhanced clinical utility.

## Data Availability

All relevant data generated or analysed during this study are included in this published article and its supplementary information files.
